# Diagnostic potential of hypoxia-induced genes in liquid biopsies of breast cancer patients

**DOI:** 10.1038/s41598-021-87897-2

**Published:** 2021-04-22

**Authors:** Carlos Henrique F. Peiró, Matheus M. Perez, Glauco S. A. de Aquino, Jéssica F. A. Encinas, Luiz Vinícius de A. Sousa, Glaucia Luciano da Veiga, Auro del Giglio, Fernando L. A. Fonseca, Beatriz da Costa Aguiar Alves

**Affiliations:** 1 Laboratório de Análises Clínicas do Centro Universitário Saúde ABC, Faculdade e Medicina do ABC, Av. Lauro Gomes, 2000, Santo André, SP CEP 09060-870 Brazil; 2 Laboratório de Epidemiologia e Análise de Dados, FMABC, Santo André, SP Brazil; 3 Departamento de Oncologia e Hematologia do Centro Universitário Saúde ABC-Faculdade de Medicina do ABC, Santo André, SP Brazil; 4 Instituto de Ciências Farmacêuticas, Universidade Federal de São Paulo (UNIFESP), Diadema, SP Brazil

**Keywords:** Molecular biology, Transcription

## Abstract

In tumor cells, higher expression of glucose transporter proteins (GLUT) and carbonic anhydrases (CAIX) genes is influenced by hypoxia-induced factors (HIF).Thus, we aimed to study the expression profile of these markers in sequential peripheral blood collections performed in breast cancer patients in order to verify their predictive potential in liquid biopsies. Gene expressions were analyzed by qPCR in tumor and blood samples from 125 patients and 25 healthy women. Differential expression was determined by the 2(−ΔCq) method. Expression of *HIF-1α* and *GLUT1* in the blood of breast cancer patients is significantly higher (90–91 and 160–161 fold increased expression, respectively; p < 0.0001) than that found in healthy women. Their diagnostic power was confirmed by ROC curve. CAIX is also more expressed in breast cancer women blood, but its expression was detected only in a few samples. But none of these genes could be considered predictive markers. Therefore, evaluation of the expression of HIF-1α and GLUT1 in blood may be a useful laboratory tool to complement the diagnosis of breast cancer, in addition to being useful for follow-up of patients and of women with a family history of breast cancer.

## Introduction

Breast neoplasms, the leading cause of cancer mortality among women, is a clinically heterogeneous disease and 10–15% of patients present an aggressive disease with distant metastasis in the first 3 years after detection of the primary tumor^1^. Tumor progression is characterized by rapid cell growth accompanied by alterations in the tumor cell microenvironment^2^ that requires high bioenergetic expenditure^3^. The most efficient cellular energy generation process is glucose metabolism^4^. Therefore, adequate supply of oxygen and glucose is essential for cellular functioning. The main regulator of glucose transport in tumor cells is GLUT^5^. This family is composed of 14 members, which exhibit tissue specificity to different hexoses, and at least one GLUT protein is expressed in each of the different human cell types^6^. GLUT1 is ubiquitously expressed and is responsible for the basic cellular supply of glucose.

Notwithstanding the adequate uptake of glucose, not all cells have the adequate supply of oxygen at their disposal in order to function. Hypoxia is the term used to describe oxygen deficiency in some tissues^7^. To survive hypoxia, cells either develop mechanisms to restore adequate tissue oxygenation, or adapt to new physiological conditions by switching to anaerobic metabolism. The Warburg Effect is known as aerobic glycolysis, in which even in the presence of oxygen, glucose is converted to lactic acid. Thus, while some tumor cells exhibit oxidative phosphorylation, most of the glucose uptake by these cells (approximately 66%) is metabolized by fermentation, a process ten times faster than the complete oxidation of this molecule^8^. Therefore, hypoxia-enhanced glycolysis is seen as an essential component of tumor malignancy and is considered a hallmark of invasive tumors, in addition to protecting the tumor against conventional treatment methods, indicating a worse prognosis^7^.

Hypoxia-Inducible Factors (HIF) are a family of hypoxia-activated transcription factors that regulate the effects of cellular oxygen sensors and a series of genes encoding glycolytic pathways^9^. The change in the expression of glycolytic proteins mediated by HIF causes the metabolic reprogramming of the tumor cell allowing its survival under hypoxic conditions and resistance to chemo and radiotherapeutic treatments^10,11^. Clinically, hypoxia and hypoxia-induced factors (HIF) are associated with increased distant metastases and worse prognosis in several tumors^12^.

Under hypoxia, HIF-1 activation depends on its α subunit, which is sensitive to oxygen tension. Increased gene expression of *HIF-1* (*HIF-1α* and *HIF-1β* complex) is known to be present in 53% of all types of cancer, and in 56–76% of breast cancers^13^. In addition to activating the production of enzymes of the glycolytic pathway, activation of *HIF*s leads to an overexpression of glucose transporters, accounting for the increased uptake of glucose by tumor cells^14^.

Another gene activated by *HIF-1* is *CAIX* (Carbonic anhydrase isoform IX)^15–18^. CAs are metalloenzymes responsible for the reversible conversion of CO_2_ into HCO_3_ in the presence of water^19,20^. So far, 15 CAs isoforms have already been described^20^, but among then, only isoforms IX and XII are associated with cancer. *CAIX* encodes an isoenzyme whose increased expression has been seen in several tumors but is rarely expressed in normal tissues^21^. CAIX is essential for the elimination of acidic products of glycolysis, which promote acidification of the extracellular tumor microenvironment and facilitate the survival and growth of these cells^18,22^. The increased expression of this gene, directly linked to the cellular hypoxia, with tumoral expression restricted to hypoxic or perinecrotic regions, suggests a role in the process of adaptation of the tumor to low O_2_ tensions, and is associated with worse prognosis and with chemoresistance^22^.

In breast cancer patients, detection of tumor cells spread from the primary tumor (circulating tumor cells; CTC) in peripheral blood has been associated with reduced general and progression-free survival. CTC detection and characterization techniques have the potential to play the role of “liquid biopsy” that will allow clinicians to track tumor development/progression and determine the best treatment for that patient^23^. Furthermore, it has previously been shown that circulating tumor cells have a tumor-independent response to hypoxia conditions^24^.

On account of the above, the aim of this study was to evaluate the expression of *HIF-1α*, *GLUT1* and *CAIX* in peripheral blood samples taken from breast cancer patients as well as in samples of tumor tissue and peripheral blood from healthy donors.

## Material and methods

### Subjects

To evaluate the expression of *HIF-1α*, *GLUT1* and *CAIX* and their association with pathological variables such as clinical stage, hormone receptors, HER2 and recurrence, breast cancer patients from the oncology department of FMABC were included in this study. Women older than 18 years with breast cancer confirmed by pathological examination, without previous chemotherapy or radiotherapy treatment and without a history of diabetes and renal and/or cardiovascular diseases were included. Patients who did not meet the criteria for inclusion or who chose not to participate in the study were excluded. From each patient, peripheral blood samples were analyzed at diagnosis and at 3 and 6 months after initiating the chemotherapy treatment, as well as biopsy specimens of the original tumor. As a control, peripheral blood samples from healthy women (donor), older than 18 years with no history of breast neoplasia (confirmed by routine annual screening for breast cancer) or other, diabetes, renal and/or cardiovascular disease were used. All patients and healthy donors signed a Free and Informed Consent Form. This study was approved by Centro Universitário Saúde ABC/Faculdade de Medicina do ABC Ethical Committee (Approval number 2.433.922).

### Total RNA isolation

From tumor samples, two 10 µm sections per block were used to obtain RNA, using RNeasy FFPE kit (Qiagen, DE) according to manufacturer's directions. From peripheral blood, 5 mL were collected by venipuncture in an EDTA tube and RNA was obtained using TRIzol reagent (TRIzol LS Reagent, Thermo Fisher, USA) according to manufacturer's directions. The extracted material was quantified by a NanoDrop Lite Spectrophotometer (Thermo Fisher Scientific Inc.).

### cDNA synthesis

RNA samples (initial 1 μg) were converted into cDNA using the QuantiTect Reverse Transcription kit (Qiagen, DE), according to the manufacturer's protocol.

### qPCR

*HIF-1α, GLUT1* and *CAIX* gene expressions were evaluated by real-time PCR (qPCR). Sequences of the designed primers and their amplicon characteristics are described in Table 1. To normalize the relative expression of the target genes, mean values of *RPL13a* (a ribosomal protein) gene expression were used.

**Table 1:** Characteristics of specific primers.

Gene	Nucleotide sequence (5′–3′)	Amplicon (bp)
*HIF-1α*	F-gtgtaccctaactagccgagg	227
	R-ggctgtgtcgactgaggaaa	
*GLUT1*	F-cccagaaggtgatcgaggag	201
	R-ccagcaggttcatcatcagc	
*CAIX*	F-ctttgccagagttgacgagg	205
	R-ttggaagtagcggctgaagt	
*RPL13a*	F-ttgaggacctctgtgtatttgtcaa	126
	R-cctggaggagaagaggaaagaga	

The initial standardization of real-time PCR amplifications was performed in an Applied Biosystems 7500 Real Time PCR Systems thermocycler (Applied Biosystems, USA) in a final volume of 15 μL containing: 1 × SYBR Green mix (Quantitec SYBR Green PCR kit, Qiagen, DE), 10 pmol of each specific primer and 2 μL cDNA (initially diluted 10 times). The cyclic parameters consisted of an initial hotstart step at 95 °C for 10 min, followed by 40 repetitions of 95 °C for 15 s and 60 °C for 25 s. The calibration curve for each gene under study was made using serial dilutions of cDNA synthesized from 1 μg of mRNA from the MCF7 (tumorigenic), MCF-10A (non-tumorigenic) and MDA-MB-231 (metastatic) cell lines.

Expression of the genes studied in the different biological matrices was determined using the formula 2^(−ΔCq)^^25^.

### Statistical analysis

Qualitative variables were presented as absolute and relative frequency. For the quantitative variables with no normal distribution of the data, medians and 25 and 75% percentiles, respectively were used; as for variables with normal distribution (Shapiro–Wilk, p > 0.05) was used to describe mean, standard deviation, minimum and maximum.

To evaluate the behavior (expression) of the genes proposed in breast cancer patients and donors (healthy women = control group), association of the diagnostic variables with the markers from the first collection was studied using the Mann–Whitney test. Finally, the Friedman test was performed to analyze the evolution of *HIF* and *GLUT1*. For all analyzes, a confidence level of 95% was used. The program utilized was Stata version 11.0 and GraphPad Prism version 6.0.

All methods were carried out in accordance with relevant guidelines and regulations.

### Ethics approval

This work was approved by Centro Universitário Saúde ABC/faculdade de Medicina do ABC Ethical Committee (Approval number 2.433.922). All methods were carried out in accordance with relevant guidelines and regulations.

## Results

In order to study the expression of *HIF-1α, GLUT1* and *CAIX* genes in liquid biopsy of patients with breast cancer, samples from 125 patients and 25 healthy donors were evaluated. As state before, healthy donors were women without a history of neoplasias, diabetes and renal and/or cardiovascular diseases. The mean age of donors was 50 y.o. (50 ± 8 y.o.), pairing with patients. The clinical characteristics of breast cancer patients are described in Table 2.

**Table 2:** Clinical characteristics of the patients.

Clinical Characteristics	n	%
**Clinical stage**		
0/I	36	28.8
II	59	47.2
III	30	24.0
**Recurrence**		
Negative	113	91.9
Positive	10	8.1
**Estrogen receptor**		
Negative	31	25.2
Positive	92	74.8
**Progesterone receptor**		
Negative	51	41.5
Positive	72	58.5
**Her_ihc**		
Negative	28	22.9
+/3+	39	32.0
++/3+	24	19.7
+++/3+	31	25.4

	Median (p.25; p.75)	Min.; Max
Age	52.5 (47; 61)	27; 78
Follow-up time (months)	28 (24.5; 32)	4; 49

+/3+, ++/3+ or +++/3+ are standard positivity index. p.25; p.75: 25–75; *Min.* minimum, *Max.* maximum.

**Figure 1:**
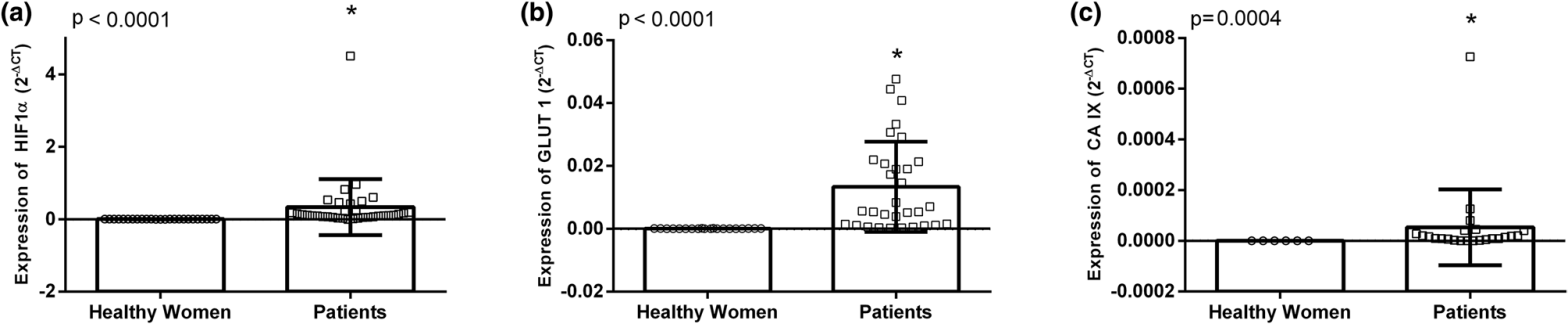
Graphic representation of the difference in expression of HIF-1α (**a**), GLUT1 (**b**) and CAIX (**c**) genes between peripheral blood samples from healthy donors and breast cancer patients (Mann–Whitney test, *CI95%* confidence interval of 95%). Gene expression was accessed by 2^−ΔCt^ formulae before the beginning of the treatment.

*HIF-1α* and *GLUT1* gene expressions were detected in all the tested samples (blood and tumor). Cq mean for *HIF-1α* was 22.5 (± 2.1) and TM was 79.3 °C; for *GLUT1*, Cq mean was 27.2 (± 2.2) and TM, 83.6 °C; the *CAIX* gene, however, was expressed in 23/125 tumors (18.4%), in 23/125 blood samples collected at diagnosis (18.4%), 24/125 blood samples collected after 3 months of chemotherapy (19.2%) and in 13/125 blood samples collected after 6 months of treatment (10.4%), with a Cq mean of 35.2 (± 2.5) and TM of 85.0 °C. Cq mean for reference gene was 19.0 (± 2.3) and TM, 78.9 °C. To verify the diagnostic potential of the expression of the target genes, a comparison was made between the mean expression of these genes in the peripheral blood of donors (healthy women) and in breast cancer patients at the time of diagnosis (Fig. 1). In breast cancer patients, expression of *HIF1-α* showed a 90–91-fold increased expression, whereas *GLUT1*, a 160–161-fold increased expression, approximately (*p* < 0.0001). Although statistically significant, the difference in expression of CAIX between the two groups was smaller and, as its expression in the samples is not consistent, this gene was left out of further analysis.

**Figure 2:**
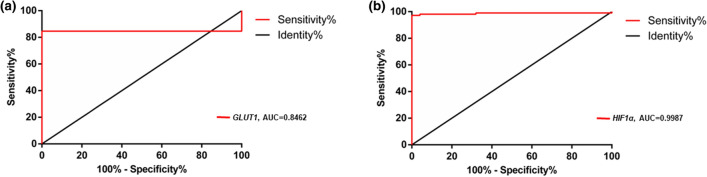
Analysis of the ROC curve to evaluate the accuracy of GLUT1 (**a**) and HIF-1α (**b**) gene expression detection in the blood as a predictive marker for the diagnosis of breast cancer. For GLUT1, AUC = 0.8462 (95% CI 0.7808–0.9115; p < 0.0001), cut-off value of 2^−ΔCq^ > 0.00017, sensitivity of 84.62% (95% CI 76.78–90.62%) and specificity of 92.31% (95% CI 74.87–99.05%); for HIF-1α, AUC = 0.9987 (95% CI 0.9683–1007; p < 0.0001)], with the cut-off value of 2^−ΔCq^ > 0.004938, with a sensitivity of 98.15% (95% CI 93.47–99.77%) and a specificity of 96% (95% CI 79.65–99.90%). Gene expression was accessed by 2^−ΔCq^ formulae before the beginning of the treatment.

A ROC Curve for *HIF-1α* and *GLUT1* was generated (Fig. 2). The *HIF-1α* marker presented the largest area under the curve [0.9987 (95% CI 0.9683–1007; p < 0.0001)], with the cut-off value of 2^−ΔCq^ > 0.004938, with a sensitivity of 98.15% (95% CI 93.47–99.77%) and a specificity of 96% (95% CI 79.65–99.90%). The *GLUT1* marker, on the other hand, had an area under the curve of 0.8462 (95% CI 0.7808–0.9115; p < 0.0001), cut-off value of 2^−ΔCq^ > 0.00017, sensitivity of 84.62% (95% CI 76.78–90.62%) and specificity of 92.31% (95% CI 74.87–99.05%).

**Figure 3:**
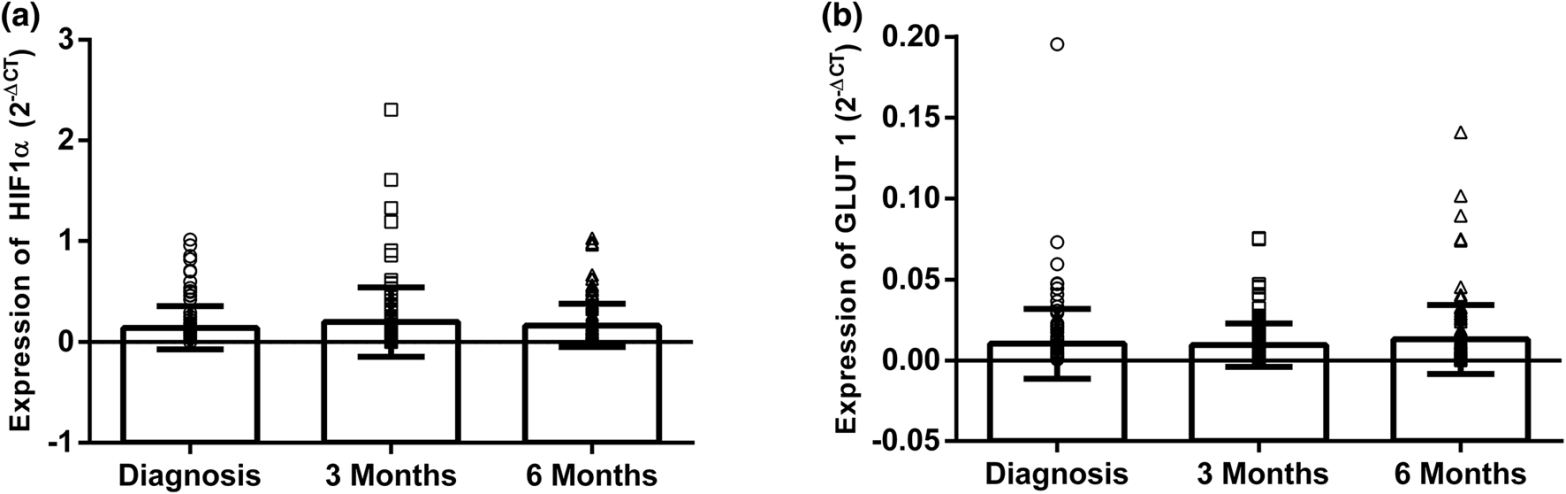
Evolution of the HIF-1α (**a**) and GLUT1 (**b**) gene expression throughout treatment (at diagnosis, 3 months after the beginning of chemotherapy and 6 months after the beginning of chemotherapy). Gene expression was accessed by 2^−ΔCq^ formula.

After verification of the diagnostic value of the differential expression of the *HIF-1α* gene in peripheral blood samples from breast cancer patients, the analysis of its potential as a predictive marker was performed. For this purpose, a comparison was made between the mean expression of this gene in the three peripheral blood collections throughout the treatment, the first being taken at the time of diagnosis, the second 3 months after the start of chemotherapy and the third, 6 months after starting treatment. The result obtained can be visualized in Fig. 3.

As mentioned previously, the *CAIX* gene was expressed in few samples (both tumor and blood). Special attention was given to the analysis of these positive expression samples; however, there was no association between expression of this marker with disease progression or clinical staging of the patient. Thus, we consider that this is not a good marker for studies in liquid biopsy. There was no relationship between the expression of the markers under study in the peripheral blood of patients with breast cancer and the other clinical characteristics (Her_ihq and hormonal receptors).

## Discussion

To date, increased expression of HIF-1^26,27^, GLUT1^28,29^ and CAIX^22^ is associated with invasion, metastasis and worse prognosis. However, to date, few studies have described the detection of the expression of *HIF-1α* and *GLUT1* genes in peripheral blood samples from patients with solid tumors^30,31^. Thus, our result confirms that peripheral blood is a suitable biological matrix for detecting the expression of these genes in patients with breast tumor. Moreover, expression of *HIF-1α* and *GLUT1* is significantly higher in peripheral blood samples from breast cancer patients than that found in healthy women, a result that points to their expression detection in this biological matrix as a potential diagnostic tool of this disease. As stated earlier, increased expression of these genes is characteristic of several tumors^27,32^, but their diagnostic potential in liquid biopsies had yet to be described.

Expression of *HIF-1α* in blood samples in normoxia has already previously been described^33,34^. The *HIF-1α* gene is expressed in CD4^+^ cells both in hypoxia and normoxia^33^ and in various cell types of the innate immune system, such as macrophages, dendritic cells, neutrophils and Th17 cells^34^. The *GLUT1* gene is ubiquitously expressed and is responsible for the basic cellular supply of glucose^6^, which justifies the detection of its expression in blood cells of healthy women. These data justify finding *HIF-1α* and *GLUT1* expressed in blood samples from healthy women. The increased expression of these genes in peripheral blood samples from breast cancer patients may be due to the presence of circulating tumor cells (CTCs). In addition, carcinogenesis is known to be closely associated with inflammatory processes^35^, and that the *HIF-1α* gene is a key metabolic reprogrammer that promotes the expression of inflammatory genes such as IL-1β^40^ and glucose transporters, data which corroborate the increased expression in cancer patients.

*CAIX* gene was expressed in few samples (both tumor and blood) of this study. *CAIX* expression is found mainly in premalignant or malignant cells, and rarely in healthy tissues or in benign lesions^36^, which justifies its detection in few samples in this study. In patients with kidney cancer, *CAIX* gene expression was detected only in 32% of blood samples^30^. Moreover, we couldn’t find any correlation between tumor and blood samples *CAIX* expression. The lack of correlation of *CAIX* expression in tumor and serum samples as well as the lack of association with clinical parameters have also been described by Schütze et al.^37^.

The ROC curve test is used to establish cut-off values for disease diagnosis, with sensitivity (ability to detect disease) and specificity (ability to minimize false-positive results) for each value obtained for the individuals in this study. The area under the curve ranges from 0 to 1 and relates to the precision of the test in the diagnosis of the disease^38^. The values generated by the ROC curve confirm the diagnostic value of the markers being studied, especially HIF-1α.

*HIF-1α* and *GLUT1* expression in peripheral blood increases significantly after initiation of chemotherapy, which indicates they can be modulated by the chemotherapeutic agent. In this study, we did not discriminate the type of chemotherapy used for each patient under consideration, since the expression profile of the markers was independent of the drug used. It was expected that with chemotherapy treatment, there would be a decrease in CTCs and thus a decrease in *HIF-1α, GLUT1* and *CAIX* expression. However, some studies have shown that even under different chemotherapy treatments, the number of CTCs varies considerably, increasing and decreasing throughout treatment^39^. This oscillation may be the reason that we have not detected alterations in gene expression throughout its treatment. Moreover, we must take into consideration that the chemotherapy treatment itself can alter the number of cells of the immune system, increasing the inflammation status, which also interferes in the expression of the genes under study.

Altered expression of *GLUT1* may influence the sensitivity of tumor cells to chemotherapy^40^. In fact, in tumor pieces, increased expression of *GLUT1* is significantly higher in patients with positive disease progression and is therefore a marker of chemoresistance^41,42^. The same is seen for *HIF-1α*^43^. Treatment of breast cancer cells with chemotherapy results in increased germ tumor cells among the surviving cells, a fact that depends on the activity of hypoxia-induced factors^44^. Thus, to assess whether there is a relationship between progression and expression of *HIF-1α* and *GLUT1*, patients were then divided according to disease progression into two groups (positive progression and negative progression). However, there was no difference in expression between these two groups. Patients were then divided according to the degree of clinical staging to assess whether the increase in gene expression is related to staging of the disease (determined at the diagnosis), however no difference in expression was found between these groups as well.

Analyzing breast tumor samples, Bos et al*.*^13^ demonstrated that HIF-1α expression is greater in samples with a more advanced stage, a result that positively associates this factor with tumor aggressiveness. In patients with acute myeloid leukemia, GLUT1 expression has also been shown to be greater in patients without remission than in patients in complete or partial remission, which results in the predictive value of GLUT1^29^. Still corroborating the role of GLUT1 as a predictive marker in a meta-analysis, Zhao et al.^44^ analyzed 41 studies with a total of 4797 patients, in whom increased GLUT1 expression was significantly associated with worse prognosis in different types of cancer. However, our results indicate that in the peripheral blood, the expression of *HIF-1α* and *GLUT1* cannot be considered as a tool for predictive evaluation or prediction of tumor aggressiveness.

Thus, our results suggest that in liquid biopsies, the differential expression of *HIF-1α* and *GLUT1* has diagnostic but not predictive potential. *CAIX*, by its turn, is also more expressed in breast cancer women than in healthy individuals. However, its expression was detected only in a few samples, so it was not included in various analyzes of this work. Therefore, evaluation of the expression of *HIF-1α* and *GLUT1* in the blood may be a useful laboratory tool to complement the diagnosis of breast cancer.

## Conclusions

The *HIF-1α* and *GLUT1* genes can be considered good markers for breast cancer diagnostic evaluations in liquid biopsies, since their expression is significantly increased in patients with excellent sensitivity and specificity values. The *CAIX* gene, however, was expressed in few samples, with no association with clinical data. The expression profile of the markers under study is compatible with the characteristics presented by the included cell lines, which technically and functionally validates the study of these genes.

## Data Availability

Data will be made available under reasonable request.
